# An Ancient Science to Improve Today’s Clinical Practice: Oral Surgery Meets Human Anatomy

**DOI:** 10.3390/ijerph182211915

**Published:** 2021-11-13

**Authors:** Roberto Pistilli, Lorenzo Bonifazi, Carlo Barausse, Alessandra Ruggeri, Michele Covelli, Maryia Karaban, Pietro Felice

**Affiliations:** 1 Oral and Maxillofacial Surgery, San Camillo-Forlanini Hospital, 00152 Rome, Italy; r_pistilli@libero.it; 2 Oral Surgery, Department of Biomedical and Neuromotor Sciences, University of Bologna, 40125 Bologna, Italy; lorenzo.bonifazi@studio.unibo.it (L.B.); carlo.barausse2@unibo.it (C.B.); maryia.karaban@studio.unibo.it (M.K.); 3 Postgraduate School of Oral Surgery, University of Modena and Reggio Emilia, 41124 Modena, Italy; 4 Human Anatomy, Department of Biomedical and Neuromotor Sciences, University of Bologna, 40100 Bologna, Italy; alessandra.ruggeri@unibo.it; 5 Centro Interuniversitario di Ricerca “Popolazione, Ambiente e Salute” (CIRPAS), University of Bari, 70124 Bari, Italy; michele.covelli@gmail.com

**Keywords:** dissection, human anatomy, oral surgery, nerve to the mylohyoid, lingual nerve, local anesthesia

## Abstract

Human body dissection was a ubiquitous practice in the past, to better understand anatomy and to develop medicine. Today, its role could still be important to answer everyday clinical queries and help surgeons. The example of the possible lack of anesthesia during symphysis surgeries can emphasize the usefulness of dissection. The mandibular symphysis usually receives innervation from inferior alveolar nerve terminations, but, in some rare cases, a particular anastomosis involves the lingual nerve and the nerve to the mylohyoid. The anatomical knowledge resulting from body dissections could help oral surgeons to understand the reason why the patient could feel pain during the surgery, and ensure performance of the right lingual nerve block to obtain complete anesthesia. This clinical situation shows the educational role of an ancient, yet still valid, practice, human dissection, and the importance of anatomical studies to improve surgical skills, to provide better treatment for the patient.

## 1. Introduction

The word dissection originates from the Latin words “dis” and “secare”, namely, “to separate” and “to cut”. Dissection can, therefore, be understood as the practice of dissecting an organism to analyze its internal structures, which does not include its meaning in the medical field, as the study of a human corpse for the purpose of teaching anatomy.

Human body dissection was already widely practiced in ancient Greece, while in Roman times, it was forbidden, so, as reported by Galen’s writings, anatomical studies continued on animals, assuming their anatomical similarity [1]. Remaining in the European context, the progressive awareness of the usefulness of human dissection as an irreplaceable cognitive and didactic method of understanding anatomy resulted in the legalization of this practice in around the fourteenth century. In the present time, anatomical dissection plays a key role in the study of anatomy, being present in many of the major medical schools worldwide [2,3]. The study of anatomical preparation represents not only an important didactic opportunity for students and neophytes, but it is useful in many medical and surgical disciplines, to better understand the anatomical reasons for certain biological traits [4,5].

Even in the dental field, especially in oral surgery, questions may arise from daily clinical practice, which can be answered thanks to the study of anatomy.

## 2. Materials and Methods

**Figure 1:**
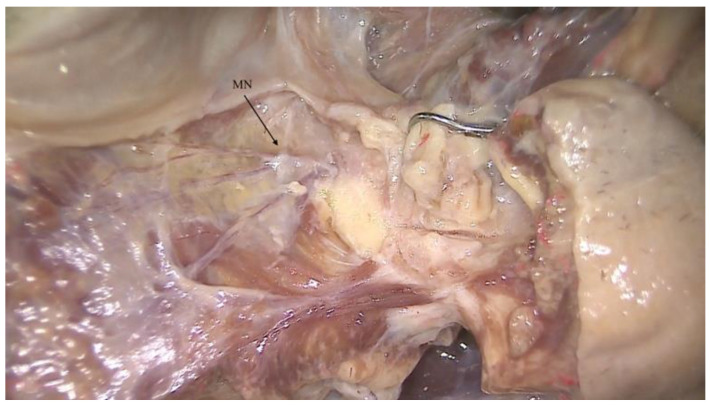
Anatomical pictures of a human body dissection showing the mental nerve fiber (MN), which is a common target of anesthesia before performing surgery in the mandibular symphysis.

**Figure 2:**
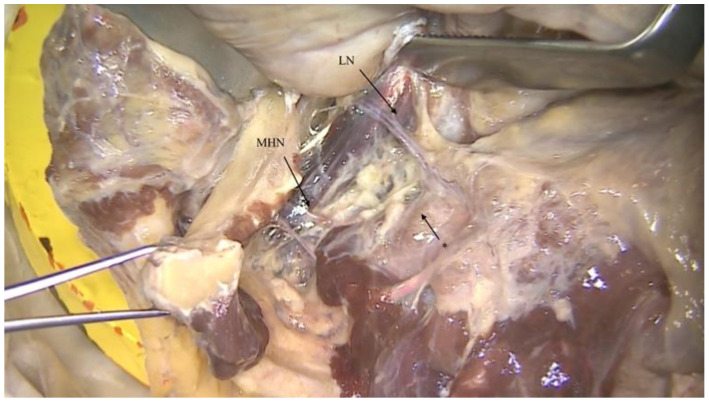
Anatomical pictures of a human body dissection showing both the nerve to the mylohyoid (MHN) and the lingual nerve (LN). The anastomosis between nerve to the mylohyoid and lingual nerve (*) can be observed.

An example of a clinical question is the possible lack of anesthesia of the mandibular symphysis during oral surgery. The mandibular symphysis is a common surgery site for the extraction or exposure of impacted lower canines, the removal of cystic lesions, the realization of mentoplasty, or for the grafting of large amounts of intraoral bone. On such occasions it is necessary to obtain complete anesthesia of the surgical area, which is classically pursued through the bilateral block of the mental nerves and possible reinforcements with local infiltrations at the symphysis (Figure 1). Sometimes, however, given that the operation is performed under local anesthesia, it is possible that the patient reports intraoperative pain despite the correct execution of the anesthetic techniques described above [6]. This possibility can trigger doubts about the success of the nerve block. These doubts, however, can be discarded thanks to an in-depth anatomical study through anatomical dissection of the lower jaw (Figure 2).

## 3. Results

### 3.1. The Nerve to the Mylohyoid

The mandibular symphysis receives terminal innervation from the third trigeminal branch (V3), via the inferior alveolar nerve. The inferior alveolar nerve then divides into the mental nerve, which emerges from the homonymous foramen at the level of the apexes of the first and second lower premolar, and in the incisor nerve, which continues its intra-bony course until it anastomoses medially with the contralateral nerve. The nerve to the mylohyoid is also found in the symphyseal area. It is a motor nerve that detaches from V3 before the mandibular nerve enters the mandible. It can originate from different distances that refer to the entrance into the mandibular foramen, between 13.4 and 14.7 mm, as reported in the literature. Once detached, the nerve runs anteriorly along the mandibular lingual surface in the mylohyoid groove. This groove can also be found in the form of an intra-bony canal, enclosing the neurovascular bundle. At the end of the mylohyoid groove, the motor fibers of the mylohyoid nerve end in the homonymous muscle, and in the anterior belly of the digastric muscle. Normally, this nerve is only responsible for muscle motility, which should not create any problems for symphysis anesthesia [7].

**Figure 3:**
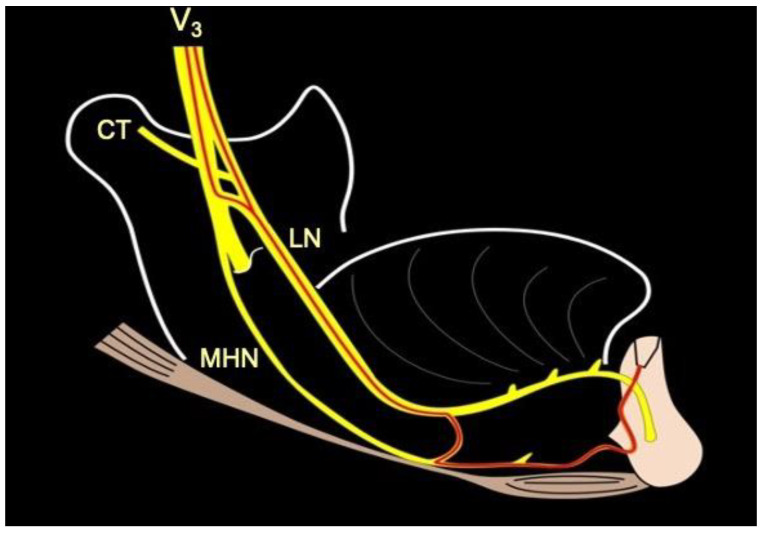
Schematic illustrating the anastomosis between the lingual nerve and the nerve to the mylohyoid. Due to this possible anatomy, the nerve to the mylohyoid could be responsible for the sensibility of the mandibular symphysis.

**Figure 4:**
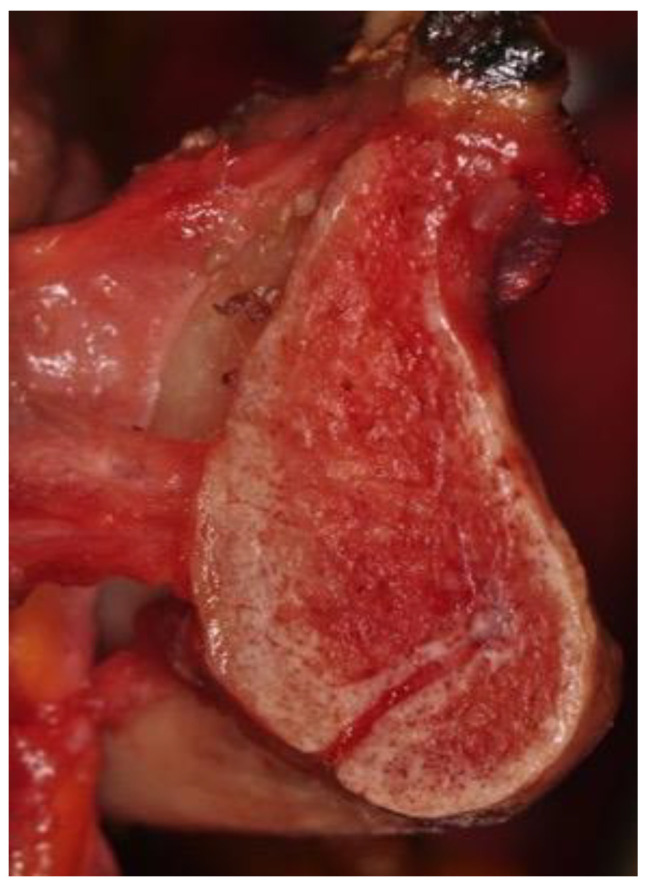
Anatomical picture of half of a mandible showing the nerve and artery to the mylohyoid entering the bone in the symphysis area.

However, it is not uncommon to find an anastomosis between the nerve to the mylohyoid and the terminations of the lingual nerve [8] (Figure 3 and Figure 4). 

### 3.2. The Lingual Nerve

The lingual nerve is also a posterior branch that detaches from the mandibular nerve, and then exits the infratemporal fossa, and passes between the tensor of the veli palatini and the lateral pterygoid muscle. After descending into the plane between the medial and lateral pterygoid muscles, it enters the pterygomandibular space between the lateral pterygoid and the mandibular branch, running more anteriorly and medially than the course of the inferior alveolar nerve. The lingual nerve continues to run along the mandibular body, in which it forms an imprint on its medial aspect below the third molar and immediately above the posterior margin of the mylohyoid line. This precise position is well known to dentists due to the risk of injury it causes during third molar surgery. The lingual nerve lies on the lateral side of the tongue, crossing the styloglossus muscle, and then traveling with the hyoglossus above the submandibular gland and its duct, with which it has an intimate relationship. The lingual nerve then passes through the mylohyoid muscle and rests on the genioglossus muscle. From here, the lingual nerve divides into several terminal branches below the tongue mucosa, providing sensory innervation of the anterior 2/3 of the tongue, the lingual floor, and the lingual gingiva of the molar and premolar area [9,10].

Crossing the duct of the submandibular gland, the lingual nerve can give rise to an unusual lateral branch that anastomoses with the nerve to the mylohyoid, usually below it, as it passes through the mylohyoid muscle [11].

**Figure 5:**
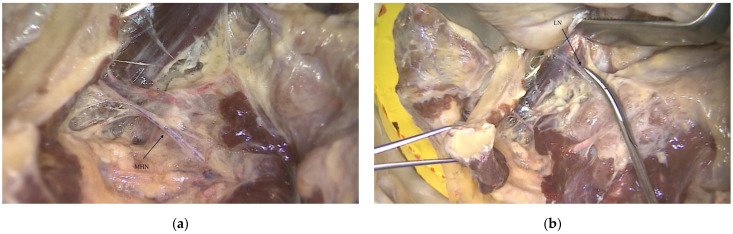

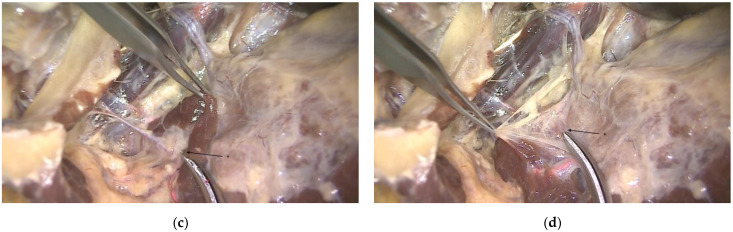
Anatomical pictures of a human body dissection, from which it is possible to better understand the intimate relation between the lingual nerve and the nerve to the mylohyoid: (**a**) nerve to the mylohyoid (MHN), (**b**) lingual nerve (LN), (**c**,**d**) anastomosis between nerve to the mylohyoid and lingual nerve (*).

In patients who have the described anastomosis, an anesthetic injection at only the level of the mental foramina may not be sufficient to obtain total anesthesia, causing painful perception during surgery. The nerve to the mylohyoid, in fact, in such cases, collects the sensitivity of the symphyseal area. The clinical solution derives from the knowledge of this anatomical variant, which is easily understood if highlighted during a cadaver dissection (Figure 5). The clinician must, therefore, proceed, in the event of sensory perception during surgery in the symphyseal area, with a bilateral anesthetic block of the lingual nerves.

The lingual nerve block has an injection point that is distal to the second molar and 6–8 mm lower than the lingual gingival margin. The insertion axis of the needle originates from the contralateral canine region and turns to the second molar. The 5–8 mm penetration of the needle will allow one to reach the target area for lingual nerve anesthesia. This procedure must be performed bilaterally to ensure adequate anesthesia of the symphysis in the above-described cases of anastomosis of the lingual nerve and the nerve to the mylohyoid [12].

## 4. Discussion

There is no doubt that this anesthetic procedure primarily involves knowledge of the possible anastomosis, the course of the lingual nerve, and the point where the lingual nerve meets the nerve to the mylohyoid. As a result, anatomical study of this region may be crucial [13].

Thus, from the clinical example of the lack of anesthesia to the mental symphysis, the importance of anatomical study, particularly through dissection, can be better understood. The knowledge deriving from anatomical dissection plays a key role, not least because of its usefulness for the patient. In the described case, in fact, the patient can benefit greatly from being able to safely receive surgery of the symphyseal area under complete analgesia, thanks to the knowledge acquired by the surgeon.

## 5. Conclusions

The study of anatomy through dissection is an ancient practice that can still be a useful tool for clinicians today, to find answers to their daily practice questions, raising their level of preparation and the quality of their work. Human body dissection is crucial not only in medical school, providing the student with a solid foundation of anatomy, but also for expert surgeons, to grant their patient a safe and high-quality clinical procedure.

## Data Availability

Not applicable.
